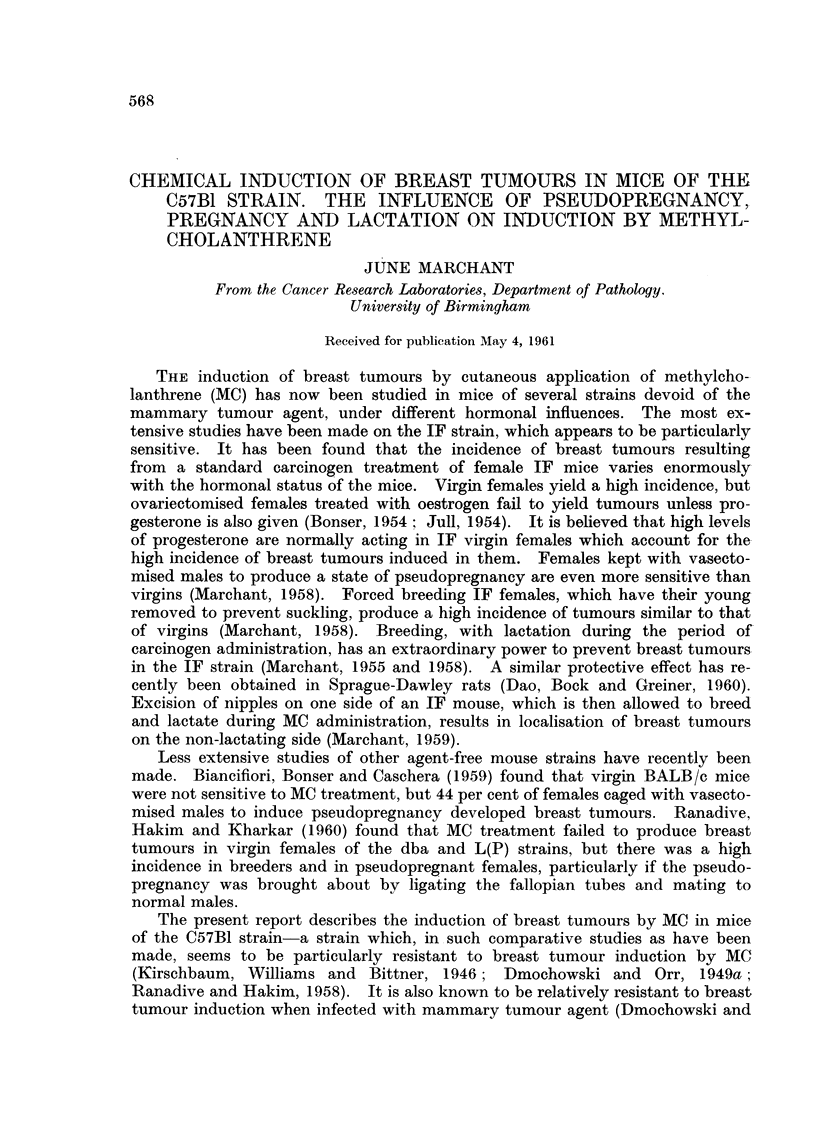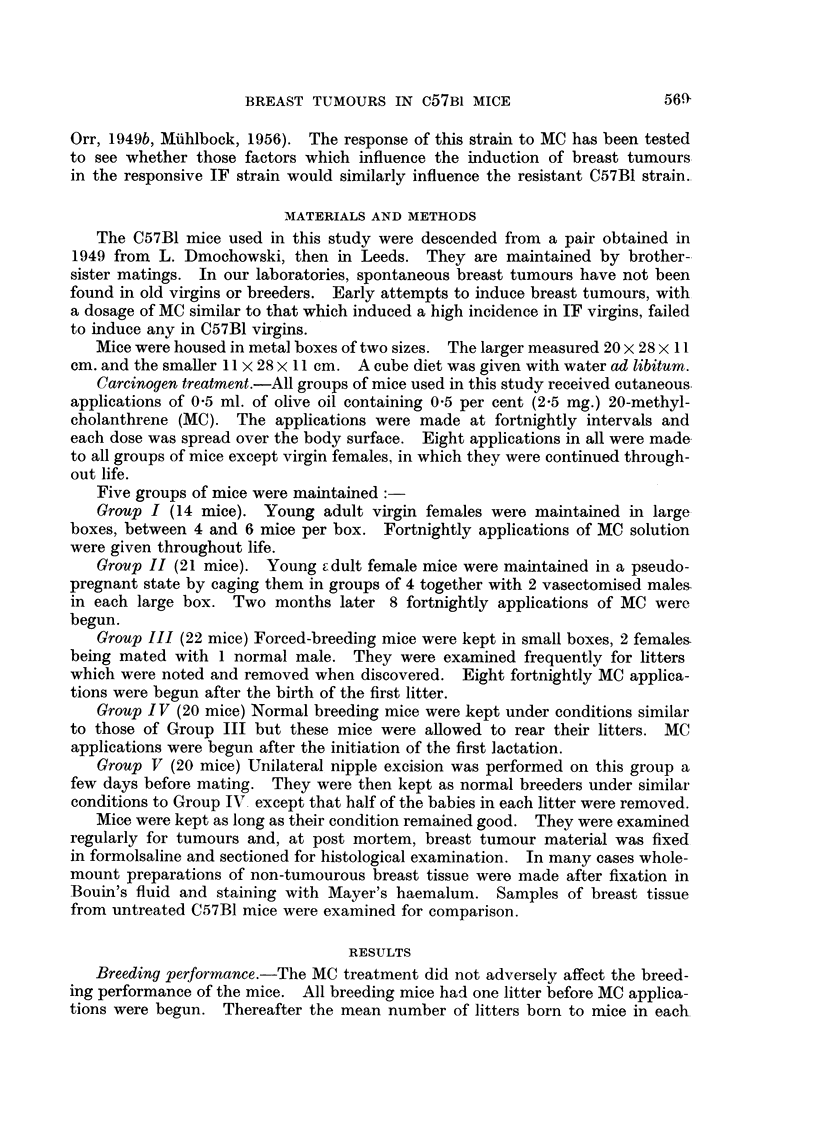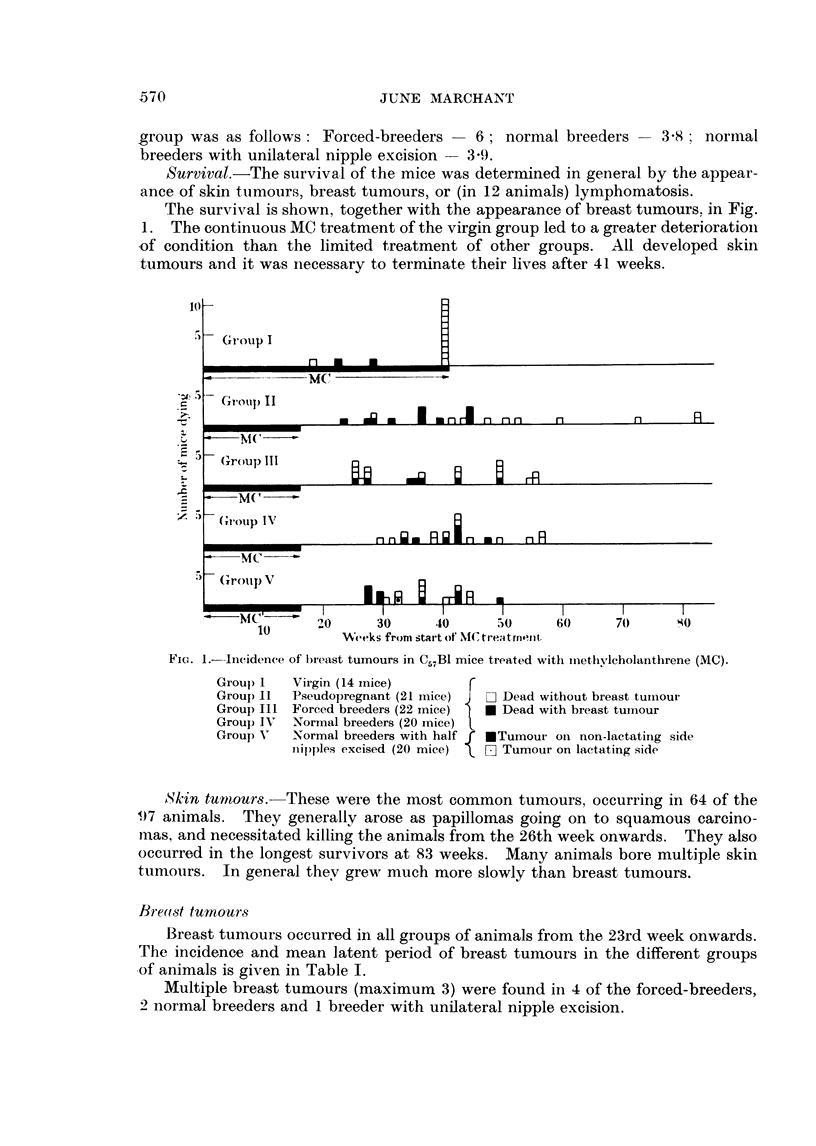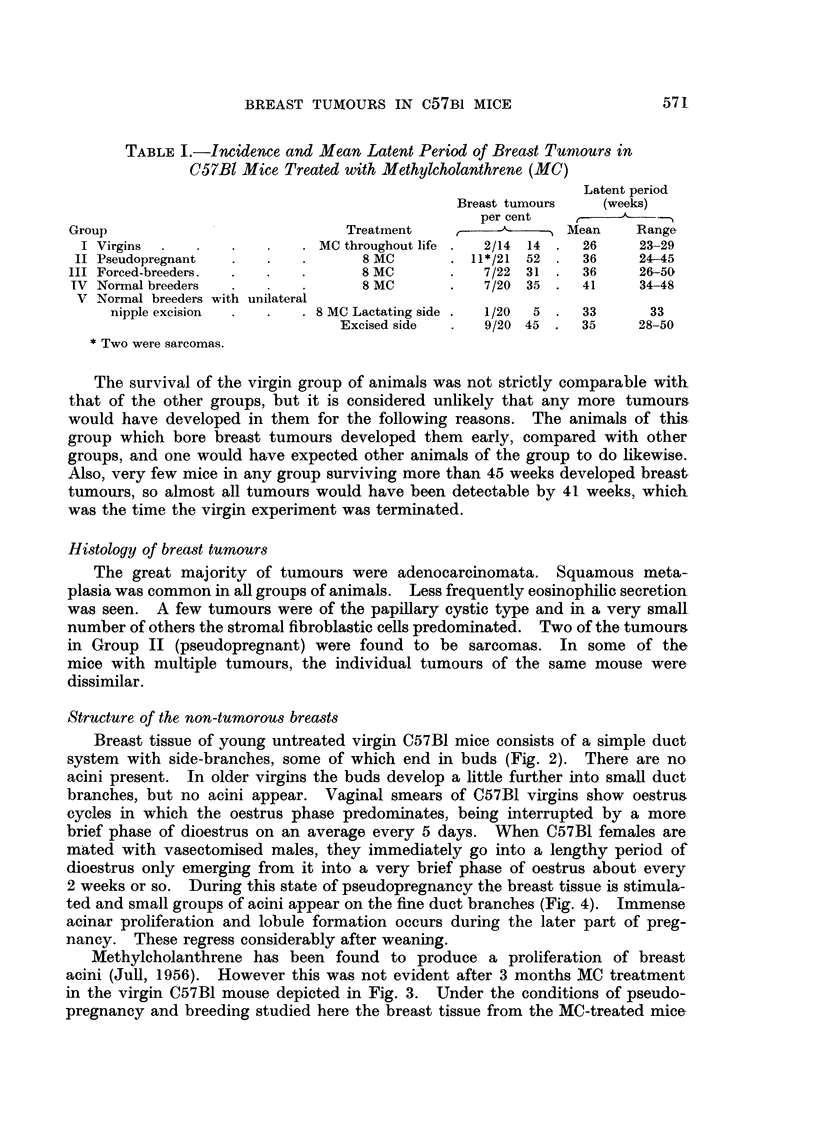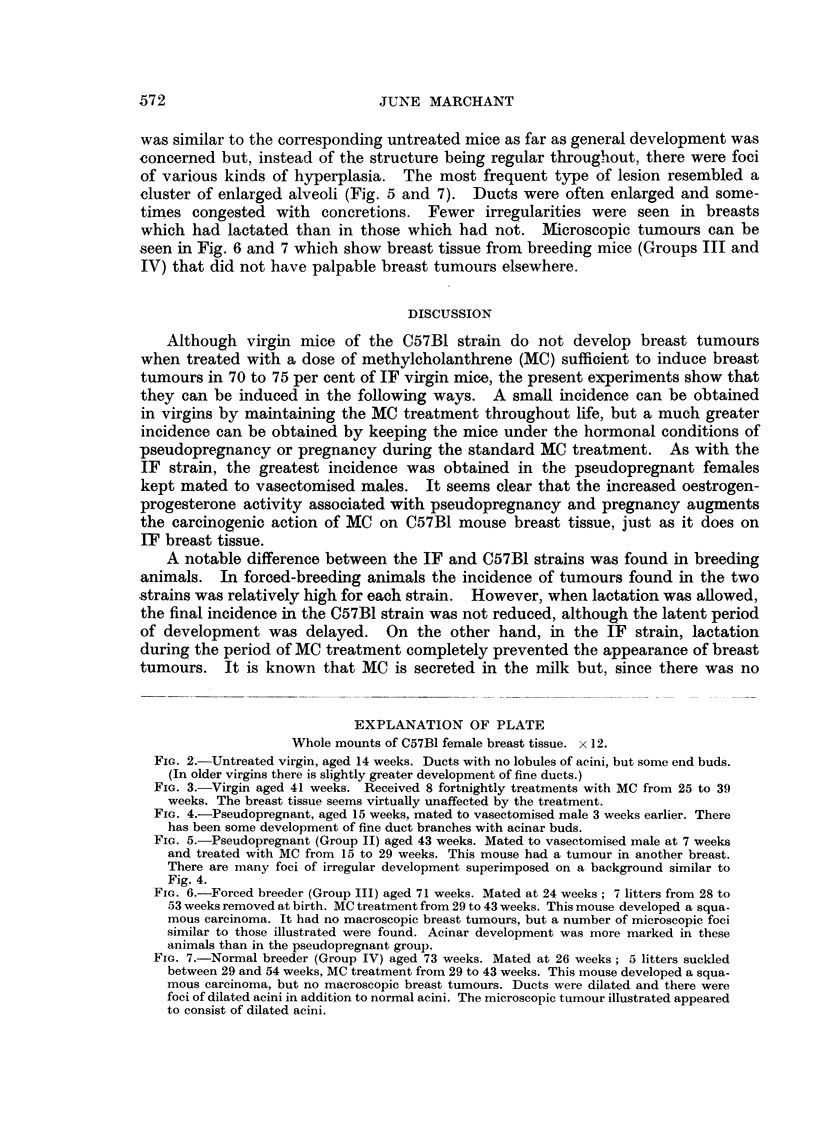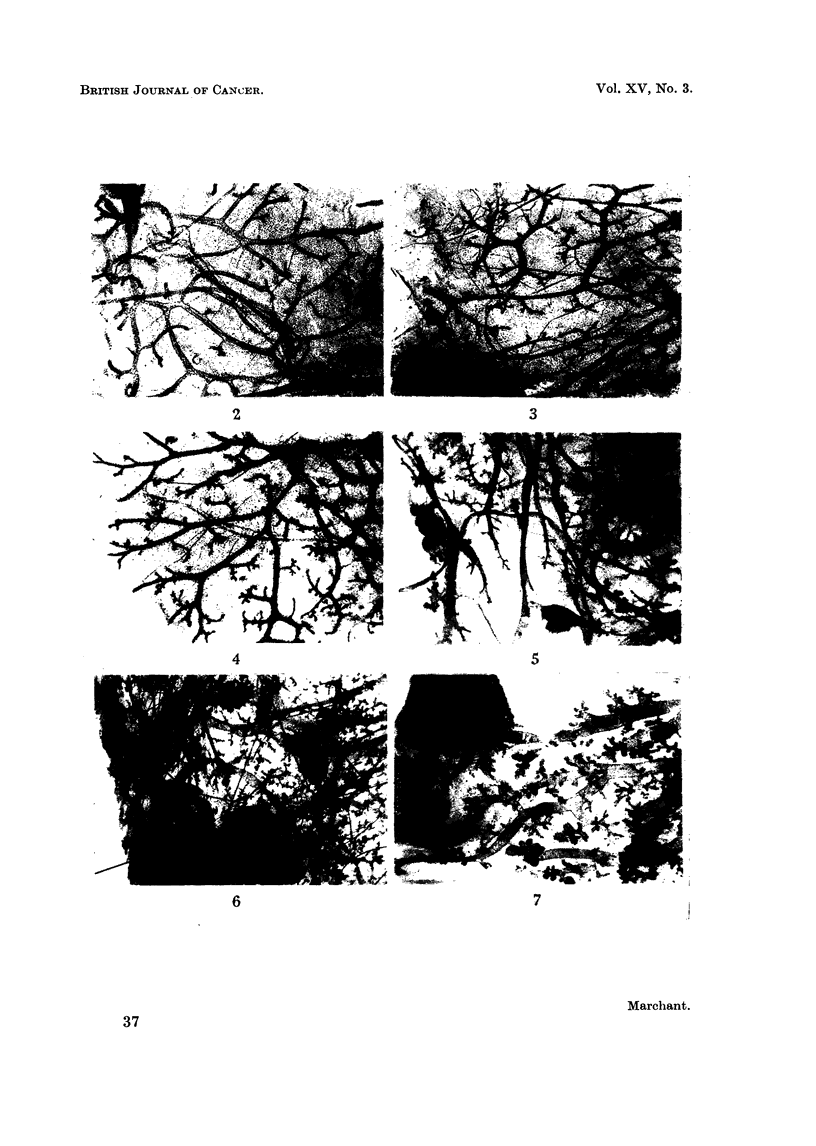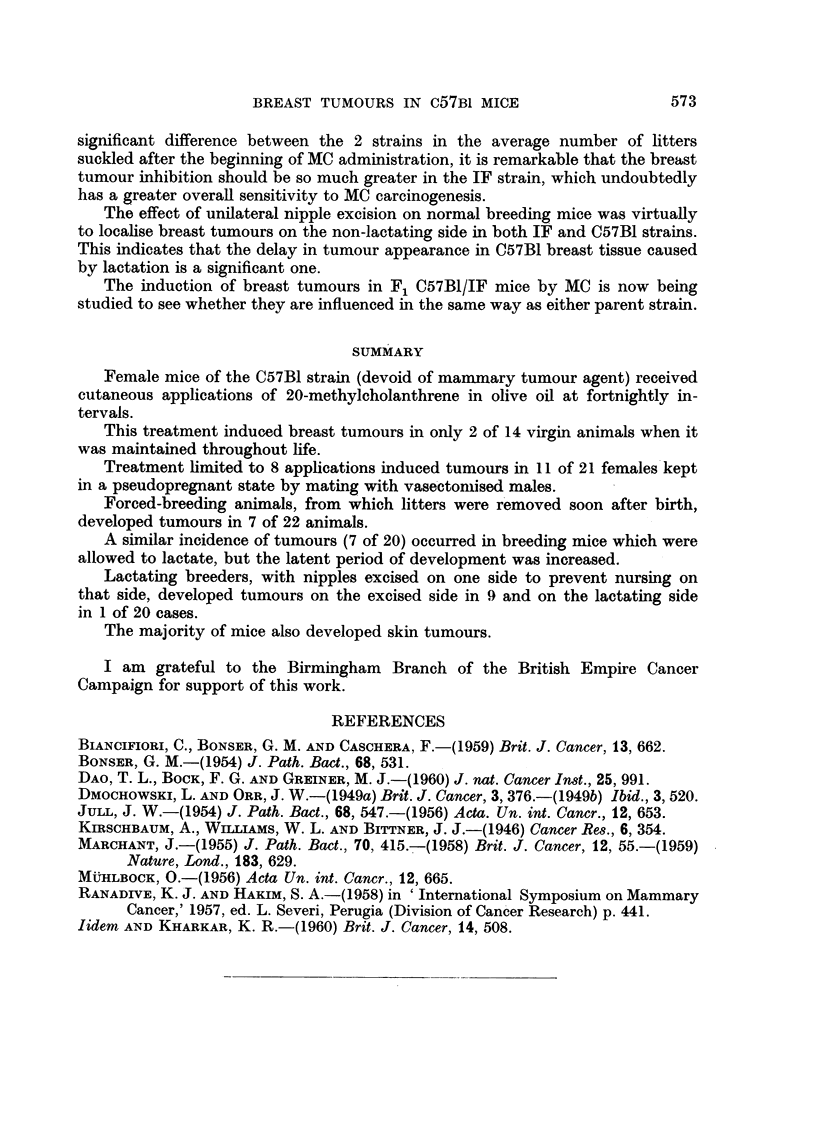# Chemical Induction of Breast Tumours in Mice of the C57Bl Strain. The Influence of Pseudopregnancy, Pregnancy and Lactation on Induction by Methylcholanthrene

**DOI:** 10.1038/bjc.1961.66

**Published:** 1961-09

**Authors:** June Marchant

## Abstract

**Images:**


### CHEMICAL INDUCTION OF BREAST TUMOURS IN MICE OF THE C57Bl STRAIN. THE INFLUENCE OF PSEUDOPREGNANCY, PREGNANCY AND LACTATION ON INDUCTION BY METHYLCHOLANTHRENE

JUNE MARCHANT

*From the Cancer Research Laboratories, Department of Pathology, University of Birmingham*

Received for publication May 4, 1961

THE induction of breast tumours by cutaneous application of methylcholanthrene (MC) has now been studied in mice of several strains devoid of the mammary tumour agent, under different hormonal influences. The most extensive studies have been made on the IF strain, which appears to be particularly sensitive. It has been found that the incidence of breast tumours resulting from a standard carcinogen treatment of female IF mice varies enormously with the hormonal status of the mice. Virgin females yield a high incidence, but ovariectomised females treated with oestrogen fail to yield tumours unless progesterone is also given (Bonser, 1954 ; Jull, 1954). It is believed that high levels of progesterone are normally acting in IF virgin females which account for the high incidence of breast tumours induced in them. Females kept with vasectomised males to produce a state of pseudopregnancy are even more sensitive than virgins (Marchant, 1958). Forced breeding IF females, which have their young removed to prevent suckling, produce a high incidence of tumours similar to that of virgins (Marchant, 1958). Breeding, with lactation during the period of carcinogen administration, has an extraordinary power to prevent breast tumours in the IF strain (Marchant, 1955 and 1958). A similar protective effect has recently been obtained in Sprague-Dawley rats (Dao, Bock and Greiner, 1960). Excision of nipples on one side of an IF mouse, which is then allowed to breed and lactate during MC administration, results in localisation of breast tumours on the non-lactating side (Marchant, 1959).

Less extensive studies of other agent-free mouse strains have recently been made. Biancifiori, Bonser and Caschera (1959) found that virgin BALB/c mice were not sensitive to MC treatment, but 44 per cent of females caged with vasectomised males to induce pseudopregnancy developed breast tumours. Ranadive, Hakim and Kharkar (1960) found that MC treatment failed to produce breast tumours in virgin females of the dba and L(P) strains, but there was a high incidence in breeders and in pseudopregnant females, particularly if the pseudopregnancy was brought about by ligating the fallopian tubes and mating to normal males.

The present report describes the induction of breast tumours by MC in mice of the C57Bl strain—a strain which, in such comparative studies as have been made, seems to be particularly resistant to breast tumour induction by MC (Kirschbaum, Williams and Bittner, 1946 ; Dmochowski and Orr, 1949*a* ; Ranadive and Hakim, 1958). It is also known to be relatively resistant to breast tumour induction when infected with mammary tumour agent (Dmochowski and

Orr, 1949*b*, Mühlbock, 1956). The response of this strain to MC has been tested to see whether those factors which influence the induction of breast tumours in the responsive IF strain would similarly influence the resistant C57Bl strain.

### MATERIALS AND METHODS

The C57Bl mice used in this study were descended from a pair obtained in 1949 from L. Dmochowski, then in Leeds. They are maintained by brother-sister matings. In our laboratories, spontaneous breast tumours have not been found in old virgins or breeders. Early attempts to induce breast tumours, with a dosage of MC similar to that which induced a high incidence in IF virgins, failed to induce any in C57Bl virgins.

Mice were housed in metal boxes of two sizes. The larger measured 20 × 28 × 11 cm. and the smaller 11 × 28 × 11 cm. A cube diet was given with water *ad libitum.*

*Carcinogen treatment.*—All groups of mice used in this study received cutaneous applications of 0·5 ml. of olive oil containing 0·5 per cent (2·5 mg.) 20-methyl-cholanthrene (MC). The applications were made at fortnightly intervals and each dose was spread over the body surface. Eight applications in all were made to all groups of mice except virgin females, in which they were continued throughout life.

Five groups of mice were maintained :—

*Group I* (14 mice). Young adult virgin females were maintained in large boxes, between 4 and 6 mice per box. Fortnightly applications of MC solution were given throughout life.

*Group II* (21 mice). Young adult female mice were maintained in a pseudo-pregnant state by caging them in groups of 4 together with 2 vasectomised males in each large box. Two months later 8 fortnightly applications of MC were begun.

*Group III* (22 mice) Forced-breeding mice were kept in small boxes, 2 females being mated with 1 normal male. They were examined frequently for litters which were noted and removed when discovered. Eight fortnightly MC applications were begun after the birth of the first litter.

*Group IV* (20 mice) Normal breeding mice were kept under conditions similar to those of Group III but these mice were allowed to rear their litters. MC applications were begun after the initiation of the first lactation.

*Group V* (20 mice) Unilateral nipple excision was performed on this group a few days before mating. They were then kept as normal breeders under similar conditions to Group IV except that half of the babies in each litter were removed.

Mice were kept as long as their condition remained good. They were examined regularly for tumours and, at post mortem, breast tumour material was fixed in formolsaline and sectioned for histological examination. In many cases whole-mount preparations of non-tumourous breast tissue were made after fixation in Bouin's fluid and staining with Mayer's haemalum. Samples of breast tissue from untreated C57Bl mice were examined for comparison.

### RESULTS

*Breeding performance.*—The MC treatment did not adversely affect the breeding performance of the mice. All breeding mice had one litter before MC applications were begun. Thereafter the mean number of litters born to mice in each group was as follows : Forced-breeders — 6 ; normal breeders — 3·8 ; normal breeders with unilateral nipple excision — 3·9.

*Survival.*—The survival of the mice was determined in general by the appearance of skin tumours, breast tumours, or (in 12 animals) lymphomatosis.

The survival is shown, together with the appearance of breast tumours, in Fig. 1. The continuous MC treatment of the virgin group led to a greater deterioration of condition than the limited treatment of other groups. All developed skin tumours and it was necessary to terminate their lives after 41 weeks.

FIG. 1.—Incidence of breast tumours in $C_{57}$Bl mice treated with methylcholanthrene (MC).

Group I Virgin (14 mice)

Group II Pseudopregnant (21 mice)

Group III Forced breeders (22 mice)

Group IV Normal breeders (20 mice)

Group V Normal breeders with half nipples excised (20 mice)

□ Dead without breast tumour

■ Dead with breast tumour

■ Tumour on non-lactating side
⊡ Tumour on lactating side

*Skin tumours.*—These were the most common tumours, occurring in 64 of the 97 animals. They generally arose as papillomas going on to squamous carcinomas, and necessitated killing the animals from the 26th week onwards. They also occurred in the longest survivors at 83 weeks. Many animals bore multiple skin tumours. In general they grew much more slowly than breast tumours.

*Breast tumours*

Breast tumours occurred in all groups of animals from the 23rd week onwards. The incidence and mean latent period of breast tumours in the different groups of animals is given in Table I.

Multiple breast tumours (maximum 3) were found in 4 of the forced-breeders, 2 normal breeders and 1 breeder with unilateral nipple excision.

TABLE I.—*Incidence and Mean Latent Period of Breast Tumours in C57Bl Mice Treated with Methylcholanthrene (MC)*

| Group | Treatment | Breast tumours per cent | | Latent period (weeks) Mean | Range |
|---|---|---|---|---|---|
| I Virgins | MC throughout life | 2/14 | 14 | 26 | 23–29 |
| II Pseudopregnant | 8 MC | 11*/21 | 52 | 36 | 24–45 |
| III Forced-breeders. | 8 MC | 7/22 | 31 | 36 | 26–50 |
| IV Normal breeders | 8 MC | 7/20 | 35 | 41 | 34–48 |
| V Normal breeders with unilateral nipple excision | 8 MC Lactating side | 1/20 | 5 | 33 | 33 |
| | Excised side | 9/20 | 45 | 35 | 28–50 |

* Two were sarcomas.

The survival of the virgin group of animals was not strictly comparable with that of the other groups, but it is considered unlikely that any more tumours would have developed in them for the following reasons. The animals of this group which bore breast tumours developed them early, compared with other groups, and one would have expected other animals of the group to do likewise. Also, very few mice in any group surviving more than 45 weeks developed breast tumours, so almost all tumours would have been detectable by 41 weeks, which was the time the virgin experiment was terminated.

*Histology of breast tumours*

The great majority of tumours were adenocarcinomata. Squamous metaplasia was common in all groups of animals. Less frequently eosinophilic secretion was seen. A few tumours were of the papillary cystic type and in a very small number of others the stromal fibroblastic cells predominated. Two of the tumours in Group II (pseudopregnant) were found to be sarcomas. In some of the mice with multiple tumours, the individual tumours of the same mouse were dissimilar.

*Structure of the non-tumorous breasts*

Breast tissue of young untreated virgin C57Bl mice consists of a simple duct system with side-branches, some of which end in buds (Fig. 2). There are no acini present. In older virgins the buds develop a little further into small duct branches, but no acini appear. Vaginal smears of C57Bl virgins show oestrus cycles in which the oestrus phase predominates, being interrupted by a more brief phase of dioestrus on an average every 5 days. When C57Bl females are mated with vasectomised males, they immediately go into a lengthy period of dioestrus only emerging from it into a very brief phase of oestrus about every 2 weeks or so. During this state of pseudopregnancy the breast tissue is stimulated and small groups of acini appear on the fine duct branches (Fig. 4). Immense acinar proliferation and lobule formation occurs during the later part of pregnancy. These regress considerably after weaning.

Methylcholanthrene has been found to produce a proliferation of breast acini (Jull, 1956). However this was not evident after 3 months MC treatment in the virgin C57Bl mouse depicted in Fig. 3. Under the conditions of pseudopregnancy and breeding studied here the breast tissue from the MC-treated mice was similar to the corresponding untreated mice as far as general development was concerned but, instead of the structure being regular throughout, there were foci of various kinds of hyperplasia. The most frequent type of lesion resembled a cluster of enlarged alveoli (Fig. 5 and 7). Ducts were often enlarged and sometimes congested with concretions. Fewer irregularities were seen in breasts which had lactated than in those which had not. Microscopic tumours can be seen in Fig. 6 and 7 which show breast tissue from breeding mice (Groups III and IV) that did not have palpable breast tumours elsewhere.

### DISCUSSION

Although virgin mice of the C57Bl strain do not develop breast tumours when treated with a dose of methylcholanthrene (MC) sufficient to induce breast tumours in 70 to 75 per cent of IF virgin mice, the present experiments show that they can be induced in the following ways. A small incidence can be obtained in virgins by maintaining the MC treatment throughout life, but a much greater incidence can be obtained by keeping the mice under the hormonal conditions of pseudopregnancy or pregnancy during the standard MC treatment. As with the IF strain, the greatest incidence was obtained in the pseudopregnant females kept mated to vasectomised males. It seems clear that the increased oestrogen-progesterone activity associated with pseudopregnancy and pregnancy augments the carcinogenic action of MC on C57Bl mouse breast tissue, just as it does on IF breast tissue.

A notable difference between the IF and C57Bl strains was found in breeding animals. In forced-breeding animals the incidence of tumours found in the two strains was relatively high for each strain. However, when lactation was allowed, the final incidence in the C57Bl strain was not reduced, although the latent period of development was delayed. On the other hand, in the IF strain, lactation during the period of MC treatment completely prevented the appearance of breast tumours. It is known that MC is secreted in the milk but, since there was no

---

### EXPLANATION OF PLATE

Whole mounts of C57Bl female breast tissue. ×12.

Fig. 2.—Untreated virgin, aged 14 weeks. Ducts with no lobules of acini, but some end buds. (In older virgins there is slightly greater development of fine ducts.)

Fig. 3.—Virgin aged 41 weeks. Received 8 fortnightly treatments with MC from 25 to 39 weeks. The breast tissue seems virtually unaffected by the treatment.

Fig. 4.—Pseudopregnant, aged 15 weeks, mated to vasectomised male 3 weeks earlier. There has been some development of fine duct branches with acinar buds.

Fig. 5.—Pseudopregnant (Group II) aged 43 weeks. Mated to vasectomised male at 7 weeks and treated with MC from 15 to 29 weeks. This mouse had a tumour in another breast. There are many foci of irregular development superimposed on a background similar to Fig. 4.

Fig. 6.—Forced breeder (Group III) aged 71 weeks. Mated at 24 weeks ; 7 litters from 28 to 53 weeks removed at birth. MC treatment from 29 to 43 weeks. This mouse developed a squamous carcinoma. It had no macroscopic breast tumours, but a number of microscopic foci similar to those illustrated were found. Acinar development was more marked in these animals than in the pseudopregnant group.

Fig. 7.—Normal breeder (Group IV) aged 73 weeks. Mated at 26 weeks ; 5 litters suckled between 29 and 54 weeks, MC treatment from 29 to 43 weeks. This mouse developed a squamous carcinoma, but no macroscopic breast tumours. Ducts were dilated and there were foci of dilated acini in addition to normal acini. The microscopic tumour illustrated appeared to consist of dilated acini.

2

3

4

5

6

7

significant difference between the 2 strains in the average number of litters suckled after the beginning of MC administration, it is remarkable that the breast tumour inhibition should be so much greater in the IF strain, which undoubtedly has a greater overall sensitivity to MC carcinogenesis.

The effect of unilateral nipple excision on normal breeding mice was virtually to localise breast tumours on the non-lactating side in both IF and C57Bl strains. This indicates that the delay in tumour appearance in C57Bl breast tissue caused by lactation is a significant one.

The induction of breast tumours in $F_1$ C57Bl/IF mice by MC is now being studied to see whether they are influenced in the same way as either parent strain.

### SUMMARY

Female mice of the C57Bl strain (devoid of mammary tumour agent) received cutaneous applications of 20-methylcholanthrene in olive oil at fortnightly intervals.

This treatment induced breast tumours in only 2 of 14 virgin animals when it was maintained throughout life.

Treatment limited to 8 applications induced tumours in 11 of 21 females kept in a pseudopregnant state by mating with vasectomised males.

Forced-breeding animals, from which litters were removed soon after birth, developed tumours in 7 of 22 animals.

A similar incidence of tumours (7 of 20) occurred in breeding mice which were allowed to lactate, but the latent period of development was increased.

Lactating breeders, with nipples excised on one side to prevent nursing on that side, developed tumours on the excised side in 9 and on the lactating side in 1 of 20 cases.

The majority of mice also developed skin tumours.

I am grateful to the Birmingham Branch of the British Empire Cancer Campaign for support of this work.

### REFERENCES

Biancifiori, C., Bonser, G. M. and Caschera, F.—(1959) *Brit. J. Cancer*, **13**, 662.
Bonser, G. M.—(1954) *J. Path. Bact.*, **68**, 531.
Dao, T. L., Bock, F. G. and Greiner, M. J.—(1960) *J. nat. Cancer Inst.*, **25**, 991.
Dmochowski, L. and Orr, J. W.—(1949*a*) *Brit. J. Cancer*, **3**, 376.—(1949*b*) *Ibid.*, **3**, 520.
Jull, J. W.—(1954) *J. Path. Bact.*, **68**, 547.—(1956) *Acta. Un. int. Cancr.*, **12**, 653.
Kirschbaum, A., Williams, W. L. and Bittner, J. J.—(1946) *Cancer Res.*, **6**, 354.
Marchant, J.—(1955) *J. Path. Bact.*, **70**, 415.—(1958) *Brit. J. Cancer*, **12**, 55.—(1959) *Nature, Lond.*, **183**, 629.
Mühlbock, O.—(1956) *Acta Un. int. Cancr.*, **12**, 665.
Ranadive, K. J. and Hakim, S. A.—(1958) in 'International Symposium on Mammary Cancer,' 1957, ed. L. Severi, Perugia (Division of Cancer Research) p. 441.
*Iidem* and Kharkar, K. R.—(1960) *Brit. J. Cancer*, **14**, 508.